# Cafeteria diet compromises natural adaptations of islet cell transdifferentiation and turnover in pregnancy

**DOI:** 10.1111/dme.15434

**Published:** 2024-09-10

**Authors:** Vaibhav Dubey, Neil Tanday, Nigel Irwin, Andrei I. Tarasov, Peter R. Flatt, R. Charlotte Moffett

**Affiliations:** ^1^ Centre for Diabetes, School of Biomedical Sciences Ulster University Coleraine Northern Ireland UK

**Keywords:** cafeteria diet, islet adaptation, pregnancy, transdifferentiation, β‐cell mass

## Abstract

**Background:**

Pancreatic islet β‐cell mass expands during pregnancy, but underlying mechanisms are not fully understood. This study examines the impact of pregnancy and cafeteria diet on islet morphology, associated cellular proliferation/apoptosis rates as well as β‐cell lineage.

**Methods:**

Non‐pregnant and pregnant Ins*1* ^Cre/+^;*Rosa26‐eYFP* transgenic mice were maintained on either normal or high‐fat cafeteria diet, with pancreatic tissue obtained at 18 days gestation. Immunohistochemical changes in islet morphology, β‐/α‐cell proliferation and apoptosis, as well as islet cell identity, neogenesis and ductal cell transdifferentiation were assessed.

**Results:**

Pregnant normal diet mice displayed an increase in body weight and glycaemia. Cafeteria feeding attenuated this weight gain while causing overt hyperglycaemia. Pregnant mice maintained on a normal diet exhibited typical expansion in islet and β‐cell area, owing to increased β‐cell proliferation and survival as well as ductal to β‐cell transdifferentiation and β‐cell neogenesis, alongside decreased β‐cell dedifferentiation. Such pregnancy‐induced islet adaptations were severely restricted by cafeteria diet. Accordingly, islets from these mice displayed high levels of β‐cell apoptosis and dedifferentiation, together with diminished β‐cell proliferation and lack of pregnancy‐induced β‐cell neogenesis and transdifferentiation, entirely opposing islet cell modifications observed in pregnant mice maintained on a normal diet.

**Conclusion:**

Augmentation of β‐cell mass during gestation arises through various mechanisms that include proliferation and survival of existing β‐cells, transdifferentiation of ductal cells as well as β‐cell neogenesis. Remarkably, cafeteria feeding almost entirely annuls pregnancy‐induced islet adaptations, which may contribute to the development of gestational diabetes in the setting of dietary provoked metabolic stress.

**What's New?**

- During pregnancy, pancreatic islet β‐cell mass expands to ensure adequate maternal and fetal insulin supply. The mechanisms underlying this islet mass expansion, and source of new β‐cells, are not fully understood.
- Pregnancy increases β‐cell mass through a combination of reduced β‐cell dedifferentiation as well as increased β‐cell proliferation, neogenesis and ductal to β‐cell transdifferentiation. Consumption of a cafeteria diet abrogates these pregnancy‐induced islet adaptations, predisposing to development of gestational diabetes.
- Dietary factors can prevent normal expansion of β‐cell mass during pregnancy. Uncovering mechanisms of β‐cell expansion during pregnancy may yield molecular targets/pathways for regenerating β‐cell mass in diabetes.

## 1 INTRODUCTION

A physiological period of marked insulin resistance frequently occurs during pregnancy, whereby the mother must supply excess insulin to maintain both maternal and fetal glucose homeostasis.^1^ The gestational increase in insulin resistance is dynamically balanced with a reversible increase in pancreatic β‐cell mass and insulin production.^2^ Importantly, β‐cell expansion, in some cases up to a doubling of initial β‐cell mass, has been noted during pregnancy in both human and animal models.^3^, ^4^, ^5^ Peaks of β‐cell proliferation and mass reported in rodents occur at gestational days 9.5–10.5 and 14.5–16.5, respectively,^6^, ^7^ corresponding to approximately 7 and 22 weeks of human gestation.^8^ When sufficient β‐cell mass expansion does not occur, this generally gives rise to gestational diabetes.

Notably, the lifetime risk of the development of type 2 diabetes is increased by up to 60% in women who have had a previous diagnosis of gestational diabetes.^9^ In accord, the coupling between glucose stimulus and insulin secretion is also disrupted in type 2 diabetes mellitus (T2DM), with increased insulin secretory output allowing for temporary normalisation of glycaemia.^10^ As with pregnancy, this adaptation is achieved by pancreatic islet hyperplasia.^11^ However, a resulting prolonged secretory stress attenuates glucose sensitivity of islet β‐cells, typically accompanied by a gradual decline in β‐cell mass that reverses the initial hyperplasia.^12^ Thus, T2DM and gestational diabetes are closely linked, sharing similar pathophysiological attributes at the level of the endocrine islet.

Furthermore, a significant risk factor for both gestational diabetes and T2DM is obesity, driven by consumption of high‐calorie diets.^13^ In good agreement, high‐fat feeding is known to disrupt female rodent reproductive function, linked to a detrimental alteration of incretin receptor expression.^14^ Indeed, glucagon‐like peptide‐1 (GLP‐1) secretion is lower in women with gestational diabetes,^15^ while short‐term exposure to a cafeteria diet in mice reduces the effects of GLP‐1 receptor activation.^16^ Thus, cafeteria feeding in rodents mimics a Western‐type diet and human hedonic eating patterns, representing a translatable obesogenic environment to study pregnancy‐induced islet changes, with relevance to gestational and other forms of diabetes associated with loss of β‐cell mass.^17^ Human obesity is rarely associated with consumption of the same food, as is the case with use of commercially available pelleted high‐fat diets in rodents. As such, the cafeteria diet more accurately mimics important sensory properties of foodstuffs, such as smell, texture and palatability, that helps drive calorie overconsumption in human obesity.^18^

In this work, we probe the interaction between the ‘lifestyle’ factor diet, through cafeteria feeding, with pregnancy in relation to the expansion of pancreatic islet β‐cells during late‐stage gestation, deemed to be associated with peak levels of islet expansion. We furthermore dissect the source of new β‐cells, utilising islet cell lineage tracing with tissue‐specific fluorescent reporters. Pregnancy and high‐fat ‘cafeteria’ feeding individually exert similar effects on islet morphology, but the combination of dietary and pregnancy metabolic stress impart opposing effects on islet β‐cell hyperplasia, the latter being attained predominantly via β‐cell proliferation and neogenesis.

## 2 MATERIALS AND METHODS

### 2.1 Diet and pregnancy

Ins*1* ^Cre/+^;*Rosa26‐eYFP* mice were bred in house within the Biomedical and Behavioural Research Unit at Ulster University, Coleraine, UK. The origin and characteristics of these transgenic mice have been described previously.^19^ Mice were housed in a temperature‐controlled environment (22 ± 2°C) on a 12‐h light/dark cycle. They were routinely maintained on standard chow (10% fat content, Trouw Nutrition, Norwich, UK) and normal drinking water ad libitum. For dietary intervention studies, female mice were kept on a high‐fat cafeteria diet (~36% fat content), comprising brie, cheddar cheese, peanuts, peanut butter, Nutella, spam meat and chocolate with 30% sucrose water^20^ for 18 days prior to mating, and continued on this diet until the end of the study. A combination of three high‐fat foods were presented to mice and rotated on a daily basis, with standard chow also available at all times. Foodstuffs were rotated on a cyclical basis, so that all foods were presented over a 3‐day period, with each foodstuff available to the mice on an equal number of occasions during the study period. While there is no definitive nutrient content that characterises a cafeteria‐fed rodent model, these diets generally have reduced fat content when compared to commercially available pelleted high‐fat diets (>45% vs. ∼35%), which more closely mimics nutrient intake patterns in human obesity.^18^ All mice ate at least part of each high‐fat foodstuff during every rotation, but we are unable to determine the exact calorie and nutrient content consumed by each individual mouse. In all experiments, age‐matched pregnant and non‐pregnant mice (*n* = 6–7 mice per group) were culled at 18 days gestation. At this point, body weights and non‐fasting plasma glucose were determined with pancreatic tissues dissected and processed for histological analysis. Blood was obtained from the tail vein and blood glucose recorded using a handheld Ascencia Contour blood glucose meter and test strips (Bayer Healthcare, Newbury, Berkshire, UK). All experiments were approved by Ulster University Animal Ethics Review Committee, conducted in accordance with the UK Animals (Scientific Procedures) Act 1986, and reported in line with the ARRIVE (Animal Research: Reporting of In Vivo Experiments) guidelines.

### 2.2 Immunohistochemistry

Mice were euthanised by lethal inhalation of CO_2_ followed by cervical dislocation. Immediately following this, pancreatic tissue was excised from pregnant and non‐pregnant mice and immediately fixed in 4% paraformaldehyde for 48 h. Following fixation, tissue samples underwent dehydration and clearing before embedding in paraffin and sectioning (5 μm) for immunohistochemistry analysis.^21^ Briefly, slides were immersed in xylene to remove wax, rehydrated in a series of ethanol washes of reducing concentration (100%–50%) followed by PBS. Antigen retrieval was achieved by immersion in heated citrate buffer (90°C, pH 6) followed by blocking in 4% bovine serum albumin. Primary antibodies, including insulin, glucagon, Ki‐67 and CK‐19, were then added followed by appropriate secondary antibodies (Table 1). Finally, slides were exposed to DAPI to identify nuclei before washing in PBS and mounting with glass coverslips. For assessing apoptosis, commercially available TUNEL staining (Roche Diagnostics, UK) was carried out following the manufacturer's guidance. Stained slides were imaged on an Olympus BX‐51 fluorescent microscope fitted with DAPI (350 nm), TRITC (594 nm) and FITC (488 nm) filters.

**TABLE 1:** Target, species, dilution and source of primary and secondary antibodies.

Target	Species	Dilution	Source
Primary antibodies			
Insulin	Mouse	1/400	Abcam; ab6995
Glucagon	Rabbit	1/100	Abcam; ab92517
Glucagon	Guinea‐pig	1/400	Raised in‐house
Ki‐67	Rabbit	1/500	Abcam; ab15580
GFP	Goat	1/500	Abcam; ab5450
CK‐19	Rabbit	1/500	Abcam; ab76539

Target Species	Host Species	Dilution	Source
Secondary antibodies			
Mouse	Donkey	1/400	Invitrogen; A‐21203
Rabbit	Donkey	1/400	Invitrogen; A‐21206
Guinea‐pig	Goat	1/400	Invitrogen; A‐11073
Goat	Donkey	1/400	Invitrogen; A‐11055

### 2.3 Image analysis

Cell^F^ imaging software (Olympus Soft Imaging Solutions) was employed to assess islet morphology as well as cellular proliferation, apoptosis, dedifferentiation, transdifferentiation and neogenesis, with blinded analysis of all images. Slides stained for insulin/glucagon were used to assess basic islet morphology and quantified on ImageJ software using a ‘closed polygon’ tool, being expressed as total islet, β‐ and α‐cell areas in μm^2^.^22^ Slides stained with TUNEL and Ki‐67 were used to assess apoptosis and proliferation, respectively, in β‐cells (insulin co‐stained) and α‐cells (glucagon co‐stained). Similarly, ductal cell transdifferentiation was quantified by assessing the percentage of CK‐19 positively stained pancreatic ductal cells co‐expressing insulin. In the transgenic Ins*1* ^Cre/+^;*Rosa26‐eYFP* mouse model, GFP‐positive islet cells are of initial β‐cell lineage,^23^ thus cells expressing both insulin and GFP are considered original β‐cells, whereas cells positive for GFP but lacking insulin are considered dedifferentiated β‐cells, with islet cells expressing insulin without GFP being regarded as β‐cells from a non‐β‐cell source. For each parameter assessed, >60 islets were analysed per treatment group.

### 2.4 Statistical analyses

Results were analysed using GraphPad PRISM (version 8), with data presented as mean ± SEM. Comparative analyses between groups were carried out using one‐way ANOVA, utilising a Bonferroni post hoc test for multiple comparisons between treatment groups. Results were deemed significant once *p* < 0.05.

## 3 RESULTS

### 3.1 Cafeteria diet increases body weight, glycaemia and impairs fertility

As anticipated, high‐fat cafeteria diet significantly increased weight gain in both non‐pregnant and pregnant Ins*1* ^Cre/+^;*Rosa26‐eYFP* mice (*p* < 0.001 and *p* < 0.05, respectively; Table 2). Consumption of this high‐fat, high‐sugar diet also increased non‐fasting glucose levels in both non‐pregnant and pregnant mice (*p* < 0.05 and *p* < 0.001, respectively; Table 2). In addition, pregnant mice maintained on the cafeteria diet presented with elevated body weight and circulating glucose when compared to standard chow‐fed pregnant mice (*p* < 0.05 and *p* < 0.001, respectively; Table 2).

**TABLE 2:** Body weight and non‐fasted blood glucose of pregnant and non‐pregnant Ins*1* ^Cre/+^;*Rosa26‐eYFP* mice maintained on a normal or cafeteria diet.

	Body weight (g)	Blood glucose (mmol/L)
Normal diet		
Non‐pregnant	22.0 ± 0.2	7.3 ± 0.3
Pregnant	31.6 ± 1.6**	8.7 ± 0.3*
Cafeteria diet		
Non‐pregnant	46.0 ± 1.5^ππ^	9.2 ± 0.2^π^
Pregnant	39.8 ± 3.1^Δ^	12.6 ± 1.5^ΔΔΔ^

*Note*: Parameters were measured at day 18 gestation in pregnant mice and equivalent in non‐pregnant mice. Values are mean ± SEM (*n* = 6–7 mice). Analysed by two‐way ANOVA with *p < 0.05, **p < 0.01 compared to respective non‐pregnant controls. ^π^ *p* < 0.05, ^ππ^ *p* < 0.01 compared to normal diet control mice. ^Δ^ *p* < 0.05, ^ΔΔΔ^ *p* < 0.001 compared to normal diet pregnant mice.

### 3.2 Pregnancy‐induced islet cell expansion and turnover rates are strikingly different in normal and cafeteria‐fed mice

**FIGURE 1:**
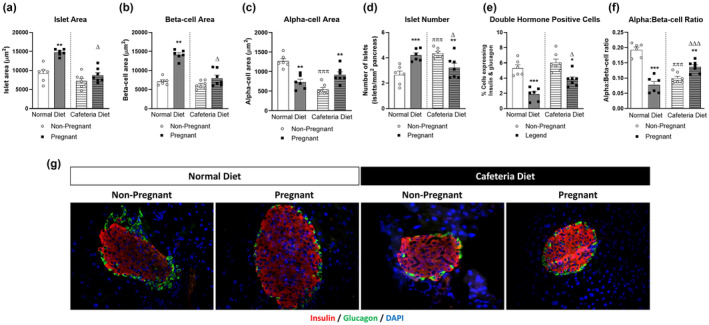
Effect of normal/cafeteria diet on islet morphology in non‐pregnant and pregnant mice. Pancreatic tissue was obtained at estational day 18 or equivalent in non‐pregnant mice. Immunohistochemistry was conducted to assess (a) islet area, (b) β‐cell area, (c) α‐cell area, (d) islet number, (e) double hormone positive cells and (f) α:β cell ratio with (g) representative images from each group showing insulin (red), glucagon (green) and DAPI (blue). Values are mean ± SEM (60 islets from *n* = 6–7 animals; at least 10 islets per mouse). Analysed by two‐way ANOVA with *p < 0.05, **p < 0.01, ***p < 0.001 compared to respective non‐pregnant controls. ^πππ^ *p* < 0.001 compared to normal diet control mice. ^Δ^ *p* < 0.05, ^ΔΔΔ^ *p* ≤ 0.001 compared to normal diet pregnant mice.

Pancreatic islet area was significantly elevated in pregnant animals maintained on a normal diet (*p* < 0.01; Figure 1a), owing to increased β‐cell area (*p* < 0.01; Figure 1b), despite a small reduction in α‐cell area (*p* < 0.01; Figure 1c). In addition, islet number was increased (*p* < 0.001; Figure 1) and the number of cells co‐expressing both insulin and glucagon decreased (*p* < 0.001; Figure 1e) in pregnant mice maintained on a normal diet. Islet morphology was largely unaltered by cafeteria feeding when compared to lean control mice, but there was a reduction in α‐cell area (*p* < 0.001; Figure 1c) and an increase in islet number (*p* < 0.001; Figure 1d). On first look, the impact of pregnancy on basic islet morphology in cafeteria‐fed mice was somewhat similar to control mice, in that both islet (Figure 1a) and β‐cell area (Figure 1b) increased, but these changes were not significant. Moreover, unlike normal diet, induction of pregnancy in cafeteria‐fed mice was associated with increased α‐cell area (*p* < 0.01; Figure 1c) and reduced islet number (*p* < 0.01; Figure 1d). Interestingly, islets from cafeteria diet‐fed pregnant mice displayed decreased islet and β‐cell areas (*p* < 0.05–0.01; Figure 1a,b) as well as reduced numbers of islets, but increased double hormone positive islet cells (*p* < 0.05; Figure 1d,e), when compared to their normal diet direct counterparts. Cafeteria feeding reduced (*p* ≤ 0.001; Figure 1f) α:β cell ratio, with similar reductions noted in normal diet pregnant mice when compared to healthy controls. In contrast, there was an increase (*p* ≤ 0.01; Figure 1f) in α:β cell ratio at day 18 gestation in pregnant cafeteria diet mice when compared to respective cafeteria‐fed control mice. Figure 1g depicts representative islet images stained for insulin (red), glucagon (green) and DAPI (blue) from each group of mice.

**FIGURE 2:**
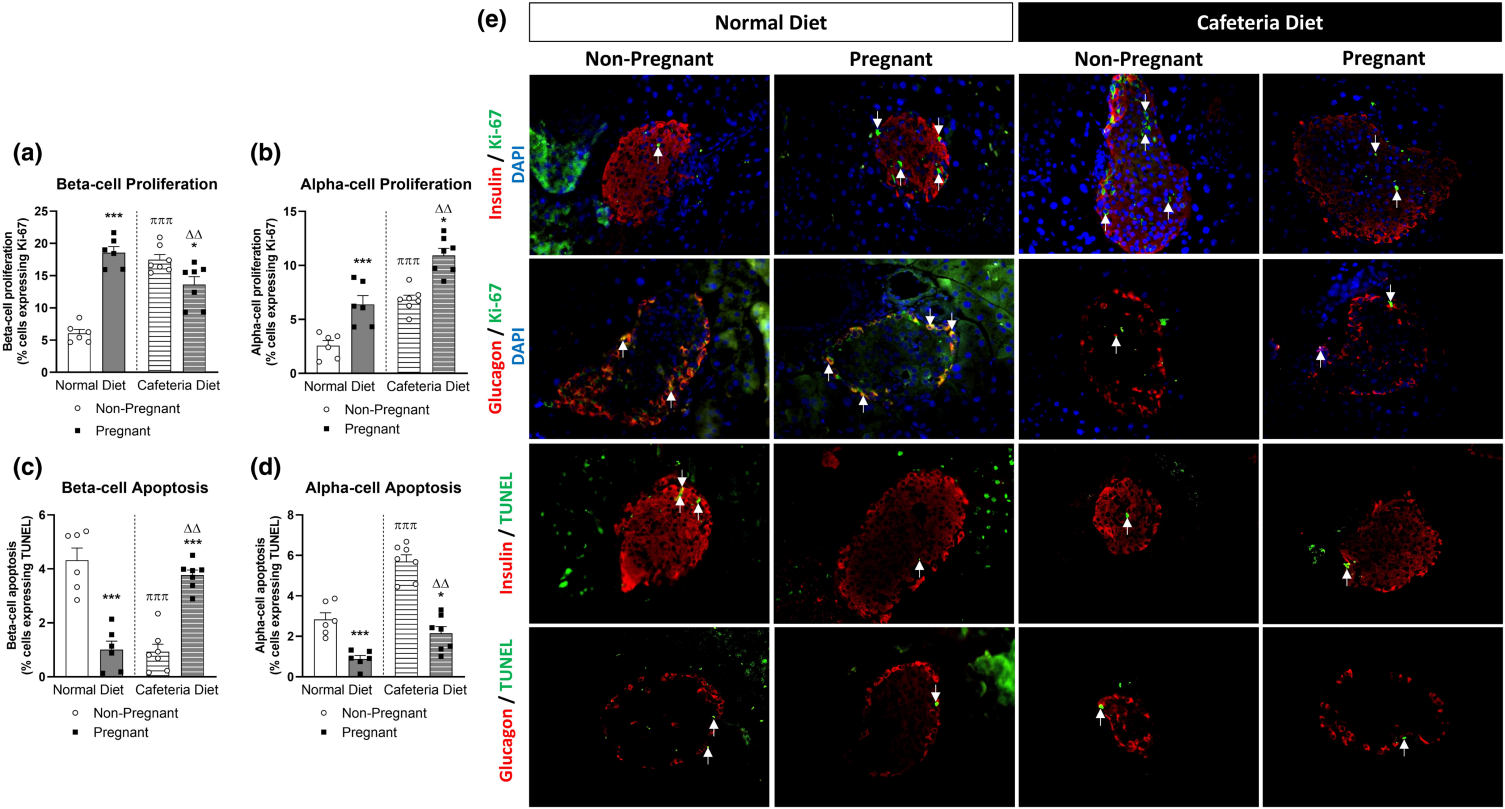
Effect of normal/cafeteria diet on β‐cell and α‐cell proliferation and apoptosis in non‐pregnant and pregnant mice. Pancreatic tissue was obtained at gestational day 18 or equivalent in non‐pregnant mice. Immunohistochemistry was conducted to assess (a) β‐cell proliferation, (b) α‐cell proliferation, (c) β‐cell apoptosis and (d) α‐cell apoptosis, (e) representative images showing DAPI (blue), Ki‐67 and TUNEL (green) staining, with arrows highlighting positive co‐staining alongside insulin or glucagon (red), as appropriate. Values are mean ± SEM (60 islets from *n* = 6–7 animals; at least 10 islets per mouse). Analysed by two‐way ANOVA with *p < 0.05, ***p < 0.001 compared to respective non‐pregnant controls. ^πππ^ *p* < 0.001 compared to normal diet control mice. ^ΔΔ^ *p* < 0.01 compared to normal diet pregnant mice.

Pregnancy induced proliferative and anti‐apoptotic effects in both α‐ and β‐cells promoted islet expansion in normal diet mice (Figure 2a–d). Remarkably, pregnancy on a background of cafeteria feeding led to opposing effects on β‐cell proliferation and apoptotic rates (Figure 2a,c), as well as α‐cell apoptosis (Figure 2d). Thus, in normal mice, pregnancy significantly stimulated the proliferation of β‐cells (*p* < 0.001; Figure 2a) as well as α‐cells (*p* < 0.001; Figure 2b). Notably, cafeteria diet alone induced proliferation of both islet cell populations in non‐pregnant animals (*p* < 0.001; Figure 2a,b). However, induction of pregnancy in cafeteria‐fed mice resulted in partial annulment of the proliferative stimulus on β‐cells (*p* < 0.05; Figure 2a), but α‐cell proliferation was further augmented (*p* < 0.05; Figure 2b). Notably, when comparing pregnant mice maintained on a cafeteria to those mice on a standard diet, there was a decrease in β‐cell proliferation (*p* < 0.01; Figure 2a) and increase in α‐cell proliferation (*p* < 0.05; Figure 2b). Further to this, pregnancy elicited a reduction in both β‐ (*p* < 0.001; Figure 2c) and α‐cell (*p* < 0.01; Figure 2d) apoptosis rates in mice fed a normal diet. Consumption of a cafeteria diet prevented pregnancy‐induced declines in β‐cell apoptosis, and somewhat unexpectedly actually elevated this parameter (*p* < 0.001; Figure 2c). In addition, while pregnant mice maintained on the cafeteria diet had reduced α‐cell apoptosis when compared to respective cafeteria‐fed controls (*p* < 0.001; Figure 2d), this was still increased when compared to pregnant mice on a normal diet (*p* < 0.05; Figure 2d). Representative islet images from each group of mice stained for insulin or glucagon (red), together with Ki‐67 or TUNEL (green), as appropriate, as well as DAPI (blue) are also provided (Figure 2e).

### 3.3 Pregnancy‐induced alterations of islet cell plasticity are severely perturbed by cafeteria feeding

**FIGURE 3:**
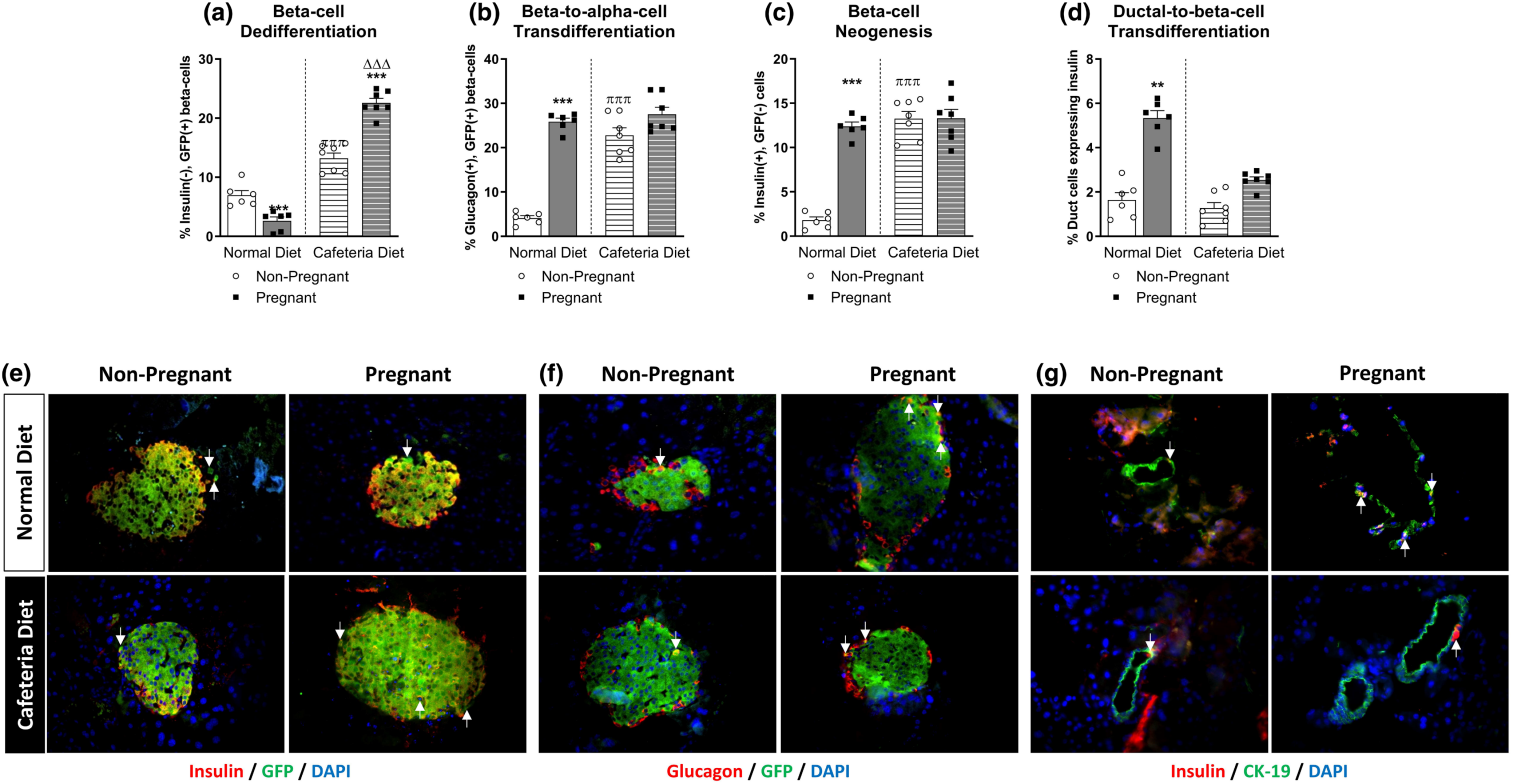
Effect of normal/cafeteria diet on β‐cell transdifferentiation and neogenesis in non‐pregnant and pregnant mice. Pancreatic tissue was obtained at gestational day 18 or equivalent in non‐pregnant mice. Immunohistochemistry was conducted to assess (a) β‐cell dedifferentiation, (b) β‐ to α‐cell transdifferentiation, (c) β‐cell neogenesis and (d) ductal to β‐cell transdifferentiation. Representative images from each group showcasing (e) insulin (red), GFP (green) and DAPI (blue), (f) glucagon (red), GFP (green) and DAPI (blue) or (g) insulin (red), CK‐19 (green) and DAPI (blue) with arrows highlighting positive co‐staining, as appropriate. Values are mean ± SEM (60 islets from *n* = 6–7 animals; at least 10 islets per mouse). Analysed by two‐way ANOVA with **p < 0.01, ***p < 0.0001 compared to respective non‐pregnant controls. ^πππ^ *p* < 0.001 compared to normal diet control mice. ^ΔΔΔ^ *p* < 0.001 compared to normal diet pregnant mice.

The percentage of dedifferentiated (YFP+insulin−) β‐cells was reduced in normal diet‐fed pregnant mice when compared to their non‐pregnant controls (*p* < 0.001; Figure 3a). At the same time, pregnancy increased the number of β‐cells transdifferentiating to α‐cells (YFP+glucagon−) (*p* < 0.001; Figure 3b) and de novo generation of β‐cells (*p* < 0.001; Figure 3c). In line with that, pregnancy significantly promoted insulin expression in CK19‐positive ductal cells (*p* < 0.01; Figure 3d). Cafeteria feeding alone altered islet cell plasticity in non‐pregnant mice, with elevations of dedifferentiated β‐cells, β‐cells transdifferentiating to α‐cells and β‐cells neogenesis (*p* < 0.001; Figure 3a–c). However, upon induction of pregnancy in cafeteria‐fed mice, other than an increase in the percentage of dedifferentiated (YFP+insulin−) β‐cells (*p* < 0.001; Figure 3a) there was no further alterations in islet cell plasticity in Ins*1* ^Cre/+^;*Rosa26‐eYFP* mice (Figure 3b–d). Figure 3e–g depicts representative islet images from each group of mice stained for insulin or glucagon (red), together with GFP or CK‐19 (green), as appropriate, as well as DAPI (blue).

## 4 DISCUSSION

In this study, we utilised a transgenic mouse model with pancreatic islet β‐cell tissue‐specific expression of a fluorescent reporter gene, YFP, to help investigate the process of islet adaptations during pregnancy.^23^ In addition, the impact of excess energy intake through Western‐style cafeteria feeding on islet‐related pregnancy outcomes was also studied.

It is now acknowledged that β‐cells can originate from a β‐, α‐ or ductal source in adulthood.^24^ In this regard, islets from pregnant Ins*1* ^Cre/+^;*Rosa26‐eYFP* transgenic mice displayed characteristic expansions in islet and β‐cell mass attributed to increased β‐cell proliferation and reduced β‐cell apoptosis.^3^, ^6^, ^7^ On further investigation, an increase in pancreatic ductal cells expressing insulin in pregnant Ins*1* ^Cre/+^;*Rosa26‐eYFP* mice was demonstrated, suggesting that non‐endocrine pancreatic cells are utilised as a source for new β‐cells during metabolic stress associated with pregnancy.^24^ Beyond ductal cells, a staggering increase in insulin‐producing cells that did not express GFP was noted in pregnant Ins*1* ^Cre/+^;*Rosa26‐eYFP* mice, raising the possibility of a non‐β‐cell endocrine origin of these cells. In keeping with this, pregnant mice exhibited a subset of GFP‐expressing β‐cells also displaying glucagon expression, indicative of a α‐ to β‐cell transformation.^24^ Whether other islet cell types represent an additional source of new β‐cells during pregnancy requires further exploration, and related investigations employing transgenic mice with α‐cell tracing capabilities would be of interest in this regard.^25^ Furthermore, in severe models of β‐cell ablation during early life, regeneration of β‐cell mass is triggered by a rapid expansion in δ‐ to β‐cell transdifferentiation.^26^

The present finding of augmented β‐cell neogenesis in pregnant mice correlates with others that observe an increase in immature (GLUT2‐deficient) β‐cells during pregnancy.^27^ These authors remark that levels of β‐cell expansion peak during E9.5, suggesting that discernible levels of islet cell transdifferentiation likely also occur during this earlier gestational time points, as well as the later day 18 stage examined here. However, it still needs to be confirmed whether these new immature β‐cells will ultimately become functional and capable of secreting insulin, with staining for markers such as Glut2, Mafa and Ucn3 potentially useful in this regard. Notably, α‐cell proliferation and apoptosis were both increased and reduced respectively. Despite these changes, overall α‐cell mass and α:β cell ratio was reduced in normal diet‐fed pregnant mice, indicating that changes in α‐cell plasticity are more relevant than related alterations of cellular turnover during classical islet adaptations to pregnancy. While it is tempting to theorise similarly for the process of β‐cell expansion in pregnancy,^7^ it is difficult to discriminate given the direction of changes in β‐cell turnover, lineage and neogenesis, all support elevated β‐cell mass. Indeed, others propose α‐cell transdifferentiation is not a major contributor to β‐cell mass expansion in pregnancy,^28^ though this study did document increased islet cell numbers in a transitional identity stage, which might suggest that the timing of observations during the gestational period are critical.

In a further series of experiments, female Ins*1* ^Cre/+^;*Rosa26‐eYFP* mice were maintained on a high‐fat, high‐sugar cafeteria diet and 30% sucrose water. Interestingly, unlike commercially available pelleted high‐fat diets which often contain a greater percentage fat content, cafeteria diet intervention had no obvious impact on overall islet morphology and β‐cell mass, barring a small reduction in α‐cell area as well as a more notable decrease in α:β cell ratio alongside an increase in islet number, the latter being observed by others.^29^, ^30^ This was despite clear elevations of body weight and blood glucose in our cafeteria diet mice. Indeed, it would have been interesting to assess body weight, glucose levels as well as all related islet parameters from each group of mice at both time of conception and 18 days gestation, but unfortunately desire not to impose associated stress at these times together with limited availability of transgenic mice did not permit for this. However, β‐cell proliferation and survival were markedly increased by cafeteria feeding,^31^, ^32^ alongside promotion of β‐ to α‐cell transdifferentiation, that together should encourage expansion of β‐cell mass as often observed with high‐fat feeding in rodents.^21^, ^33^ Thus, it is also possible that the lower fat content compared with commercially available high‐fat diets, and relatively short 18‐day period of cafeteria feeding prior to mating, precluded more robust changes in islet morphology in these mice.

Most intriguingly, when the metabolic pressure of cafeteria diet was combined with pregnancy‐related stressors, the characteristic expansions in islet morphology induced by pregnancy was abrogated, with islets failing to expand in β‐cell area or islet number, leading to a striking increase in the α:β cell ratio. In these mice, β‐cell proliferation was stunted together with increased β‐cell apoptosis, and this was accompanied by increased loss of β‐cell identity as well as inability to generate β‐cells from non‐β‐cell sources. Indeed, elevated β‐cell apoptosis and inflammation in islets from high–fat‐fed pregnant mice has been observed previously,^34^, ^35^ and these are recognised instigators of β‐cell dedifferentiation.^24^ Taken together, it appears that the triggering of neogenic β‐cell mass expansion is independently induced by cafeteria diet, and when islets are additionally stressed by pregnancy there is limited further capacity to generate new β‐cells. In keeping with this, elevated α‐cell area and proliferation in cafeteria‐fed pregnant mice may represent an adaptive response to supplement a pool of potential new progenitor β‐cells.^24^ The possibility of α‐ to β‐cell transdifferentiation postpartum would also support the importance of this process in refining β‐cell mass during pregnancy.^36^

In terms of potential underlying mechanisms, GLP‐1 receptor activation could be considered one likely candidate. This is based on knowledge that GLP‐1 receptor KO, but not KO of the receptor for the sister incretin glucose‐dependent insulinotropic polypeptide (GIP), prevents β‐cell mass expansion during pregnancy in mice^37^ and that cafeteria feeding reduces GLP‐1 activity.^16^ When this is considered alongside the fact that GLP‐1 secretion is lower in women with gestational diabetes^15^ and this incretin hormone is recognised as a key hormone in the overall control of β‐cell plasticity,^24^ it would certainly seem plausible, but it is unlikely that GLP‐1 is the sole instigator. That said, α‐cells have been shown to increase GLP‐1 synthesis and secretion relative to that of glucagon during pregnancy,^38^ which could be linked to our findings of enhanced β‐cell growth and survival through well‐described paracrine islet cell interactions.^36^ This fits well with current observations that pregnancy in normal mice increases numbers of β‐cells transdifferentiating towards an α‐cell phenotype. Either way, it is clear in the current setting that the metabolic stress brought about by the cafeteria diet was sufficient to prevent normal pregnancy‐induced islet adaptations. Failure of islet mass to expand during pregnancy can be linked to the possible development of gestational diabetes and possibly T2DM in later life.^37^ Consistent with this, non‐fasting glucose concentrations were significantly elevated at term in pregnant cafeteria‐fed mice. Although beyond the scope of this study, it would be interesting to assess the impact of maternal cafeteria feeding on fetal growth and early pup islet development, a period where islets are known to expand.^8^

## 5 CONCLUSION

This study demonstrates that the augmentation of β‐cell mass during gestation arises through proliferation and survival of existing β‐cells, reduced β‐cell dedifferentiation, transdifferentiation of pre‐existing α‐ and ductal cells and neogenesis from other non‐β‐cell sources. Remarkably, Western‐style cafeteria feeding almost entirely annuls these pregnancy‐induced islet adaptations, linked to elevated blood glucose and increased levels of β‐cell apoptosis and dedifferentiation concurrent with reduced β‐cell proliferation and failure to produce β‐cells from non‐β‐cell sources.

## AUTHOR CONTRIBUTIONS

A.I.T., N.I., P.R.F. and R.C.M. contributed to the overall concept, experimental design and interpretation of the data. N.T. and V.D. performed the experimental work and contributed to validation, formal analysis and visualisation of the data. All authors contributed to the writing the manuscript and approved the final version.

## FUNDING INFORMATION

These studies were supported by a Diabetes UK RD Lawrence Research Fellowship awarded to RCM and an Ulster University Vice‐Chancellor PhD studentship.

## CONFLICT OF INTEREST STATEMENT

The authors declare no conflict of interests.

## Data Availability

The data that support the findings of this study are available from the corresponding author upon reasonable request.
